# Smart-cut-like laser slicing of GaN substrate using its own nitrogen

**DOI:** 10.1038/s41598-021-97159-w

**Published:** 2021-09-09

**Authors:** Atsushi Tanaka, Ryuji Sugiura, Daisuke Kawaguchi, Toshiki Yui, Yotaro Wani, Tomomi Aratani, Hirotaka Watanabe, Hadi Sena, Yoshio Honda, Yasunori Igasaki, Hiroshi Amano

**Affiliations:** 1 Center for Integrated Research of Future Electronics (CIRFE), Institute of Materials and Systems for Sustainability (IMaSS), Nagoya University, Aichi, 464-8601 Japan; 2 National Institute for Materials Science, Tsukuba, 987-6543 Japan; 3 Electron Tube Division, Research & Development Department, Hamamatsu Photonics K. K., Shizuoka, 438-0193 Japan

**Keywords:** Materials science, Materials for devices, Materials for energy and catalysis

## Abstract

We have investigated the possibility of applying lasers to slice GaN substrates. Using a sub-nanosecond laser with a wavelength of 532 nm, we succeeded in slicing GaN substrates. In the laser slicing method used in this study, there was almost no kerf loss, and the thickness of the layer damaged by laser slicing was about 40 µm. We demonstrated that a standard high quality homoepitaxial layer can be grown on the sliced surface after removing the damaged layer by polishing.

## Introduction

Gallium nitride (GaN) is of particularly several applications as a wide-bandgap semiconductor material, among which light-emitting diodes (LEDs) and high-frequency devices are already in practical use. GaN is also a suitable material for power devices owing to its high critical electric field strength and high electron saturation velocity; although this application is still in the research phase, it is being actively studied^1–19^. For LEDs and high-frequency devices, the device layer of GaN crystal is grown on foreign substrate. However, the epitaxial layer grown on a GaN substrate has higher quality with lower dislocation density than that grown on a foreign substrate. To improve device quality and reliability, a GaN-on-GaN structure is required. Particularly for power device use, the epitaxial layer grown on a GaN substrate as device layer is required, because there are many reports indicating that dislocations affect the characteristics of GaN power devices^20–26^. However, a GaN substrate is more expensive than competing power device materials such as Si and SiC, and this is one of the serious barriers to its widespread adoption in power devices. To cut a GaN substrate from the GaN crystal bulk, a wire saw is used conventionally, and using a wire saw causes kerf loss greater than the diameter of the wire. Typically, the diameter of the wire saw is greater than 150 µm and the total kerf loss is greater than 200 µm. To reduce the kerf loss and cost of GaN substrates, various new slicing and thinning methods have been attempted^27–35^. However, none of them have been put to practical use. In this paper, we report on a newly developed laser slicing method for minimizing the loss of GaN substrates and demonstrate its practicality as a semiconductor processing technique.

## Experiments

**Figure 1:**
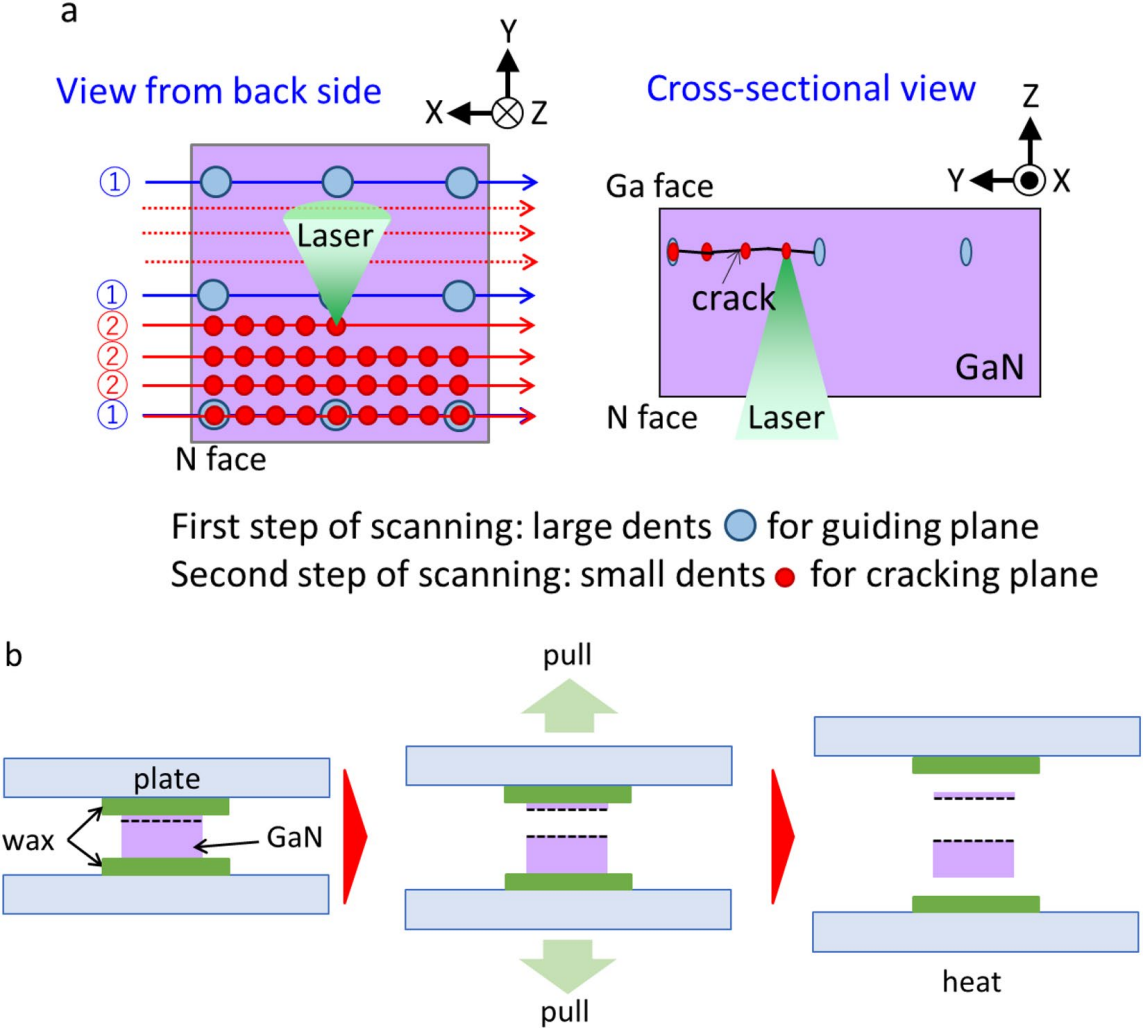
Schematic images of laser slicing. (**a**) Laser irradiation. (**b**) Separation.

As a slicing target, commercially available both-side-polished n-type wurtzite GaN (0001) c-plane substrates with a thickness of 400 µm were used in this study. A sub-nanosecond 532 nm green laser with a peak power density of 2.5 × 10^11^ W/cm^2^ and a beam diameter of 1 µm was used for slicing. This wavelength is chosen because it is not absorbed by GaN, it has sufficient photon energy that reaches the band gap of GaN through two-photon absorption and it is easy to focus and obtain. This laser was equipped with a spatial light modulator (LCOS-SLM, Hamamatsu Photonics) to compensate for the spherical aberration due to the relatively high refractive index of GaN, which is 2.4 for the light with wavelength of 532 nm^36^. This laser is irradiated from the N-face of a GaN substrate, and a flat plane was formed by laser dents at a depth of 340 µm from the N-face, imagining slicing out a 340 µm wafer. In this method, such a plane was formed by the following two steps laser scanning: The first step scanning was to form laser dents at 8 µm intervals horizontally and 10 µm intervals vertically, using a pulse energy of 1.6 µJ. The second step was to form laser dents at 1 µm intervals horizontally and vertically, using a pulse energy of 0.6 µJ. The first step of scanning was to define the plane with relatively large dents, and the second step was to crack the plane. This was to make the plane as uniform as possible, avoiding that formed metallic droplets of precipitated Ga at the laser dents affect the surrounding focus depth. Figure 1a shows schematic images of the slicing process. We have not yet worked on reducing the processing time; however, the processing speed by laser scanning was about 20 cm^2^/s. After the slicing plane was formed in the GaN substrate, the slicing process was completed by sticking each side of the GaN substrate to a jig and then pulling it away manually (Fig. 1b).

## Results and discussion

**Figure 2:**
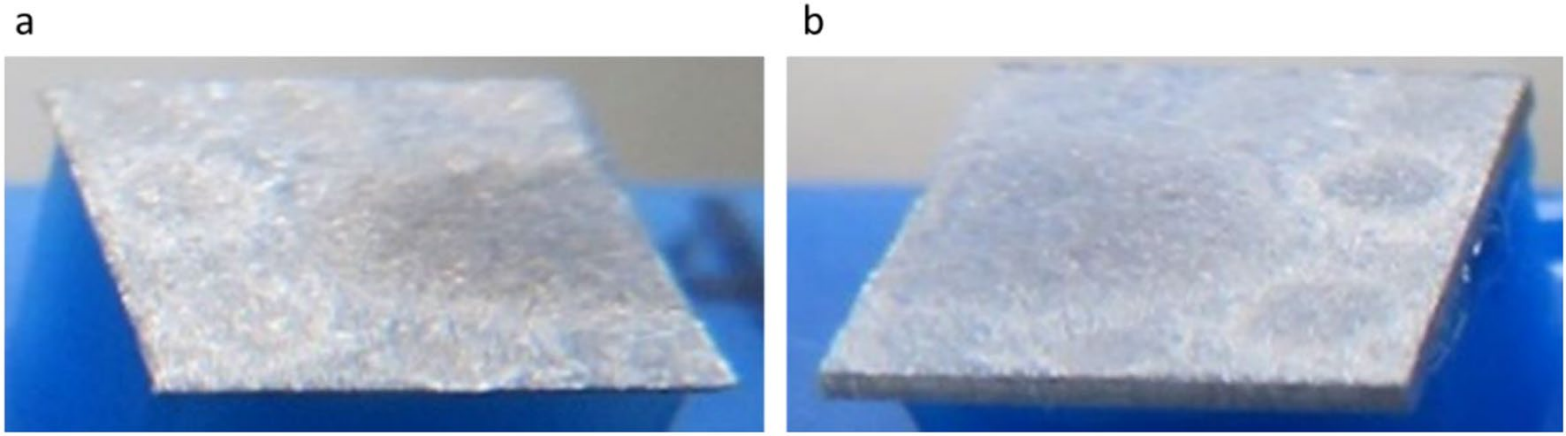
Photographs of laser-sliced 5 × 5 mm square GaN substrates. (**a**) Separated part of GaN substrate with thickness of 60 µm. (**b**) Separated part of GaN substrate with thickness of 340 µm.

Figure 2 shows photographs of a laser-sliced 5 × 5 mm square substrate. The surface of the sliced sample has a metallic luster due to the precipitated Ga, indicating that the decomposition of GaN to Ga and N in the GaN substrate is induced by laser irradiation. The density of Ga is 5.9 g/cm^3^ and the density of nitrogen is 1.3 × 10^−3^ g/cm^3^, both of which are smaller than the GaN density of 6.2 g/cm^3^. It is considered that the volume of decomposed region expands owing to the decomposition at the laser focus, and cracks are generated by pressure in the plane direction, forming the cut plane. To demonstrate this process and confirm that N_2_ plays a major role in this process, we conducted the experiment as follows.

**Figure 3:**
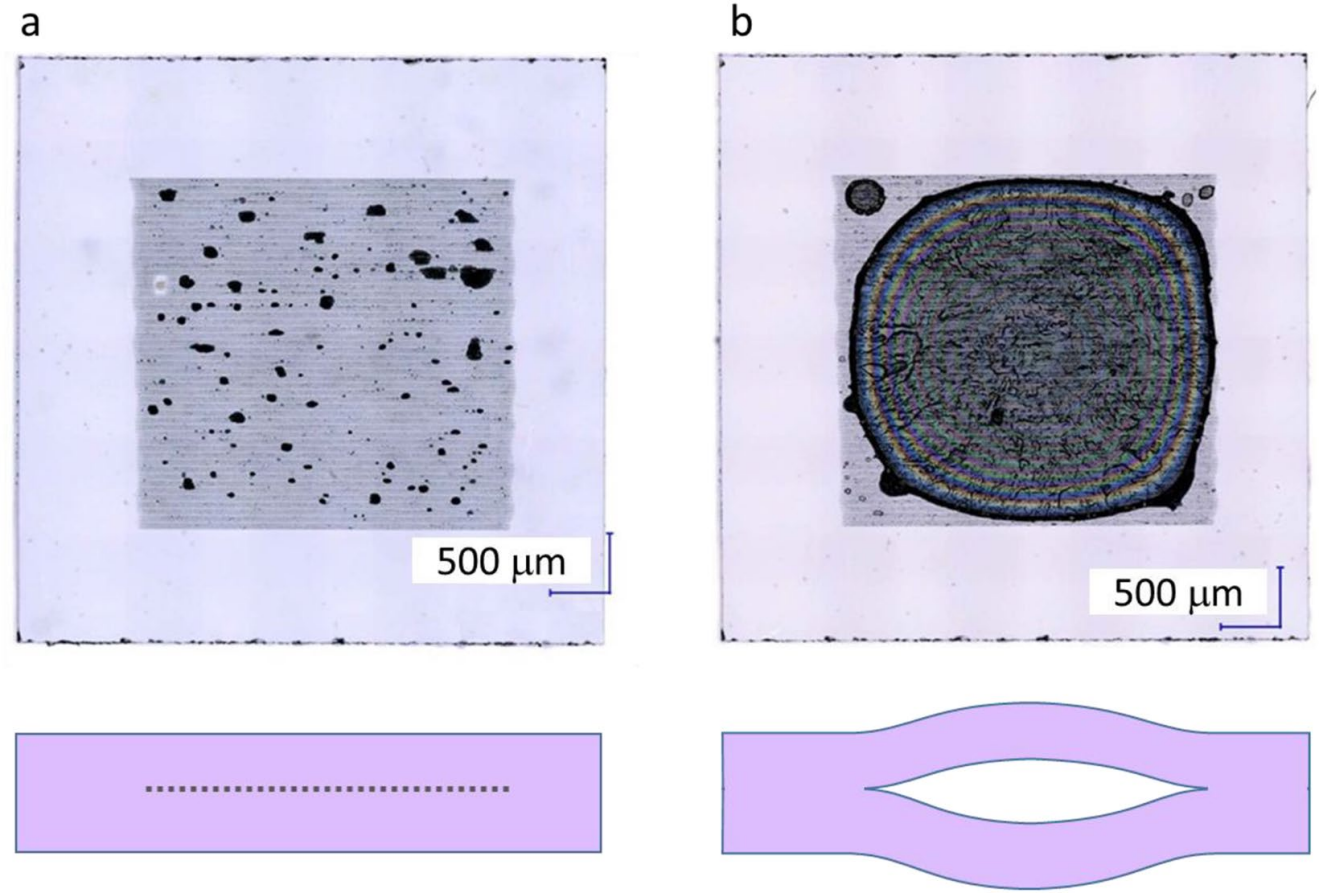
Photographs and cross-sectional schematic images of GaN substrates containing nitrogen bubbles. (**a**) After laser irradiation before heating. (**b**) After heating.

Only the first step of laser scanning was carried out at the center of the sample, leaving 700 µm of the sample periphery unscanned. At this point, there seemed to be very few Ga precipitates and the scanned area was still almost transparent (Fig. 3a). The sample was then heated to 350 °C on a hot plate. The center of the substrate swelled, and interference fringes were observed. As shown in Fig. 3b, after cooling to room temperature, there was still space inside, and interference fringes remained. This indicates that the nitrogen in the large dents expanded owing to heat and formed a separation plane. Since the photographs in Fig. 3 were taken under white LED light, assuming the central wavelength was 560 nm, the thickness of the swollen space in the GaN substrate was estimated to be about 2.5 µm at the thickest point on the basis of nine interference fringes, which means 2.52 = 0.56/2 × 9 µm. This experiment revealed that our method is a type of smart cut^32,33,37–39^, using a laser instead of an ion implantation, and using its own gas atoms, N, instead of hydrogen ions; hard and brittle GaN can withstand this process. Therefore, this result suggests the possibility of applying this method to the processing of future wide-bandgap semiconductor materials with gas atoms as components such as gallium oxide and aluminum nitride. Furthermore, since a laser is used as the cutting source instead of an ion implantation, it is possible to create a cutting plane at any depth as long as the laser can be focused.

**Figure 4:**
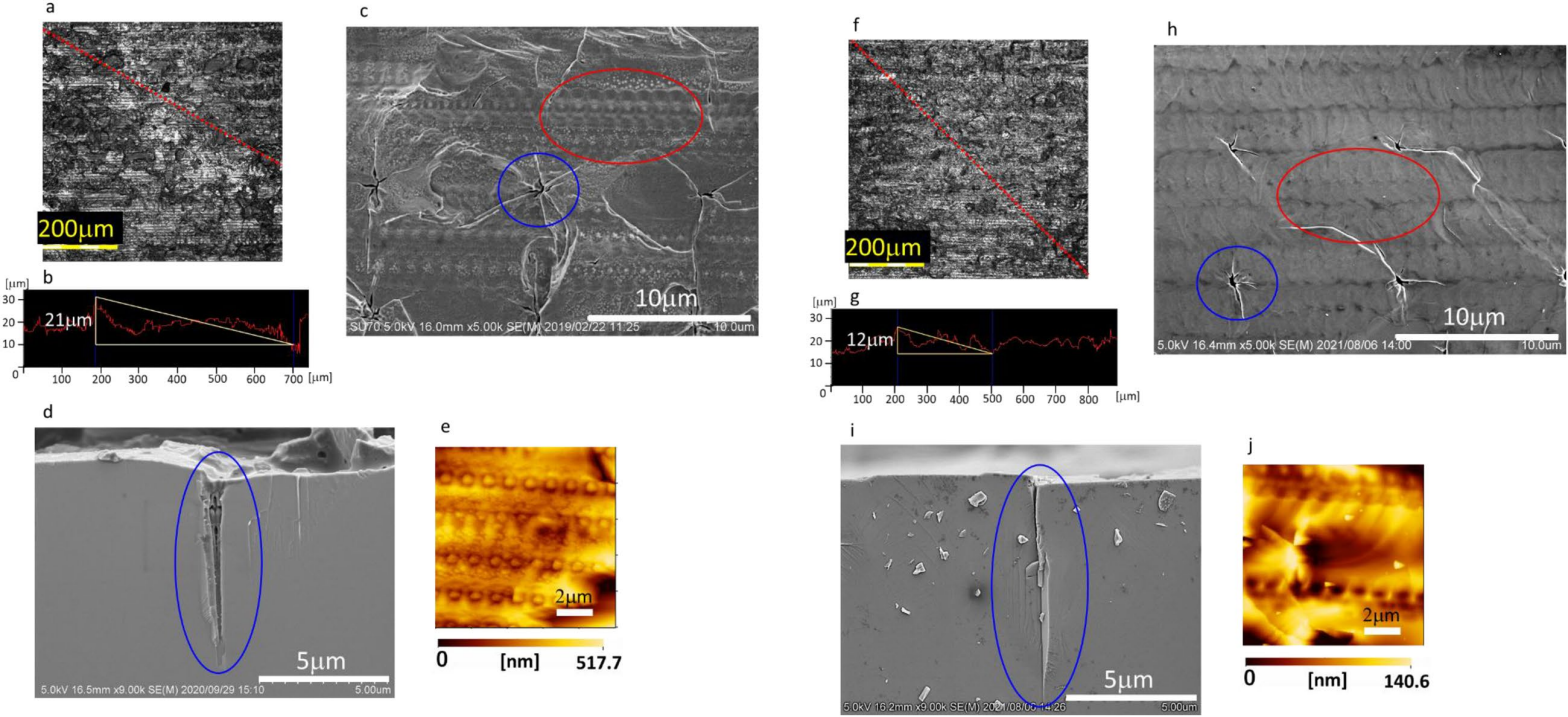
Evaluation of laser-sliced surface. (**a**–**e**) are evaluations of Ga-face of the separated part of the GaN substrate with a thickness of 340 µm. (**f**–**i)** are evaluations of N-face of the separated part of the GaN substrate with a thickness of 60 µm. (**a**,**f**) Laser microscopy image. (**b**,**g**) Height profile along red dashed line in (**a**) and (**f**). (**c**,**h**) SEM image of sliced surface. The blue circle indicates a large dent generated by the first scan. The red circle indicates small dents generated by the second scan. (**d**,**i**) Cross-sectional SEM image of large dent (blue circle). (**e**,**j**) AFM image of small dents.

Figure 4 shows laser microscopy images of the sliced surface of Ga-face (Fig. 4a,b) and N-face (Fig. 4f,g), scanning electron microscopy (SEM) images of sliced surface of Ga-face (Fig. 4c: the sliced surface, 4d: the laser dent depth) and N-face (Fig. 4h: the sliced surface, 4i: the laser dent depth), atomic force microscopy (AFM) images of sliced surface of Ga-face (Fig. 4e) and N-face (Fig. 4j) for evaluating the condition of the sliced surface of the sample shown in Fig. 2. As for the roughness of these surface, the unevenness caused by large focus fluctuations was determined to be around 10–20 µm on each side from Fig. 4a,f. The depth of large dents generated by the first scanning was determined to be around 10 µm on each side from Fig. 4d,i. The depth of small dents generated by second scanning was determined to be around 300 nm on the sliced surface of Ga-face from Fig. 4e and around 60 nm on the sliced surface of N-face. The reason why the surface morphology does not differ so much between the Ga-face and N-face in the laser microscopy images is thought to be that the laser slice is progressed by almost lateral cracks and also the shape of the large dents, which are thought to be the main cause of the roughness of the surface, does not differ between on Ga-face and on N-face. The reason why the shapes of the large dents are almost same on both sides is thought to be that the large dents are almost cracks caused by the expansion of the volume at the dents owing to the ablation of GaN. The generation of nitrogen gas is thought to be the main reason of the expansion of the volume. On the other hand, the depth of the small dents is deeper on the Ga-face than N-face. We consider this is because the Ga precipitation mainly determine the shape of the small dents, as the nitrogen can escape laterally owing to laterally spread of cracks during the second scan. In one pulse of the laser, precipitated Ga increases the absorption of the laser, and Ga precipitation extend for laser irradiation direction, which is Ga-face side of GaN substrate with thickness of 340 µm from slicing plane in this experiment. To estimate the depth of damage caused by laser slicing, three-dimensional photoluminescence (PL) observation of GaN was carried out using a multiphoton microscope^40,41^.

**Figure 5:**
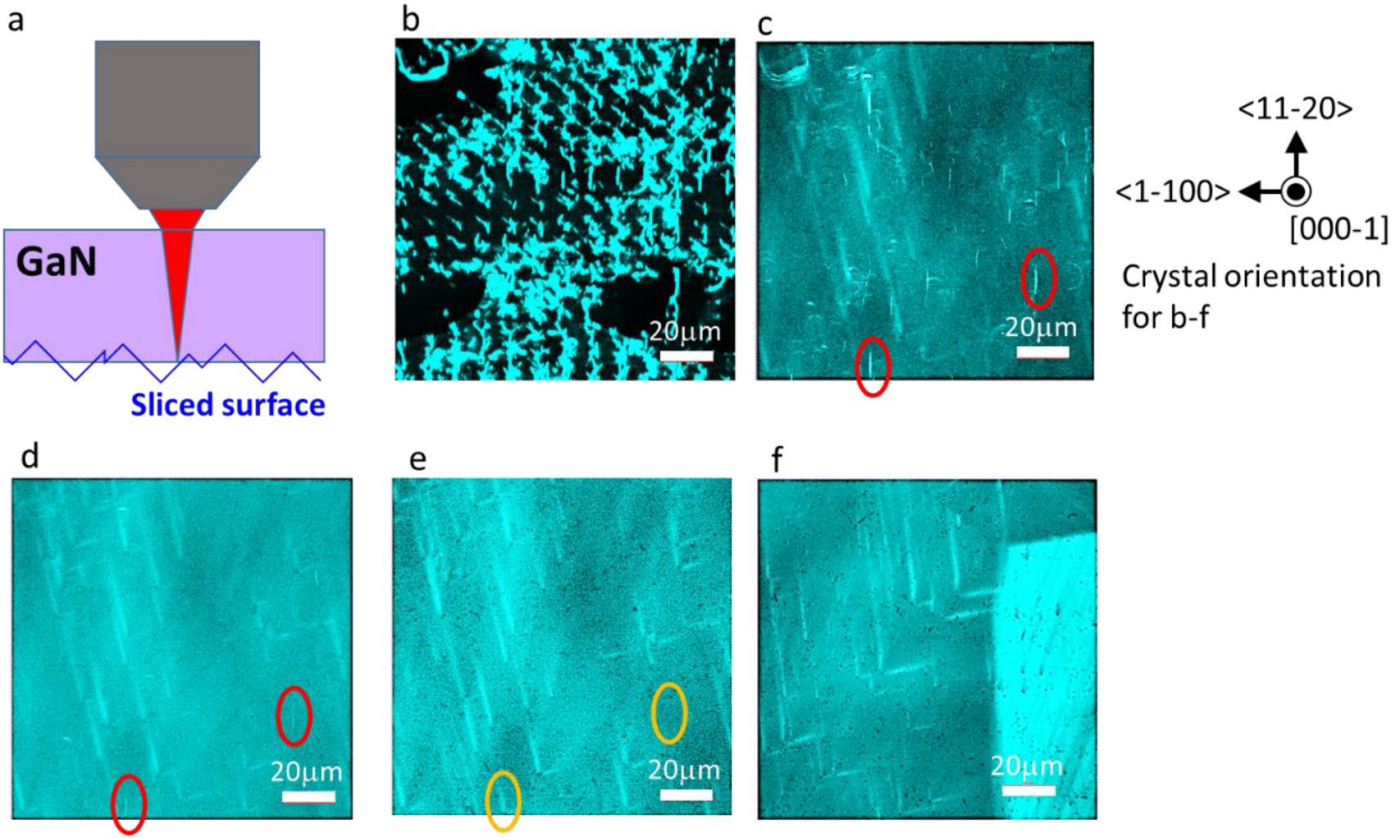
Evaluation of damage caused by laser slicing with multiphoton microscope. (**a**) Schematic cross-sectional view of multiphoton microscopy observation at the slice plane from the back side of the sample. (**b**) PL (365 nm) image observed at sliced surface. The damage generated by laser scanning is observed as a bright contrast. (**c**) PL (365 nm) image observed at a depth of 37 µm from the sliced surface (**b**). Strong linear bright contrasts show damage. Pale bright contrasts do not show damaged areas, but the area where the original properties of the substrate are slightly different, regardless of the extent of laser damage. Typical laser-damaged areas are encircled in red. (**d**) PL (365 nm) image observed at a depth of 42 µm from the sliced surface (**b**). The deep-laser-damaged area encircled in red is barely visible. (**e**) PL (365 nm) image observed at a depth of 79 µm from the sliced surface (**b**). Almost no damage contrast is visible even at the position of the deep-laser-damaged area (orange circles). Black dots are dislocation defects that originally exist in the GaN substrate. In (**b**–**d**) dislocations are difficult to recognize by multiphoton microscopy because of the depth of observation. (**f**) PL (365 nm) image of the GaN substrate that contain only substrate-derived crystal defects for comparison.

The results are shown in Fig. 5. As shown in Fig. 5b, damage at about 10 µm intervals was observed as a bright contrast around the sliced surface in near-band-edge emission (365 nm), which was probably caused by the first scanning owing to the spacing of the laser dents. The bright contrast extends linearly in the **a** direction, in which GaN easily cleaves. This suggests that the local nitrogen pressure generated by the large dent formed during the first scanning causes cracks or similar distortions in the weak direction of the GaN crystal. In addition, when large dents that are close to each other align in the **a** direction, they seem to interact with each other, and a bright contrast is observed as a line connecting the dents. In Fig. 5, the large dents are arranged in a grid as shown in Fig. 1, so the large dents lined up vertically and bright lines appeared vertically. As shown in Fig. 5b–d, this damage caused by the interaction of large dents is observed to be particularly deep. This suggests that we need to pay attention to the relationship between the arrangement of the large dents and the crystal orientation when we try to suppress the damage caused by the large dents. Regarding the depth of this damage caused by the interaction of large dents, the damage was observed at sites as far as 42 µm from the slicing plane at the deepest point.

**Figure 6:**
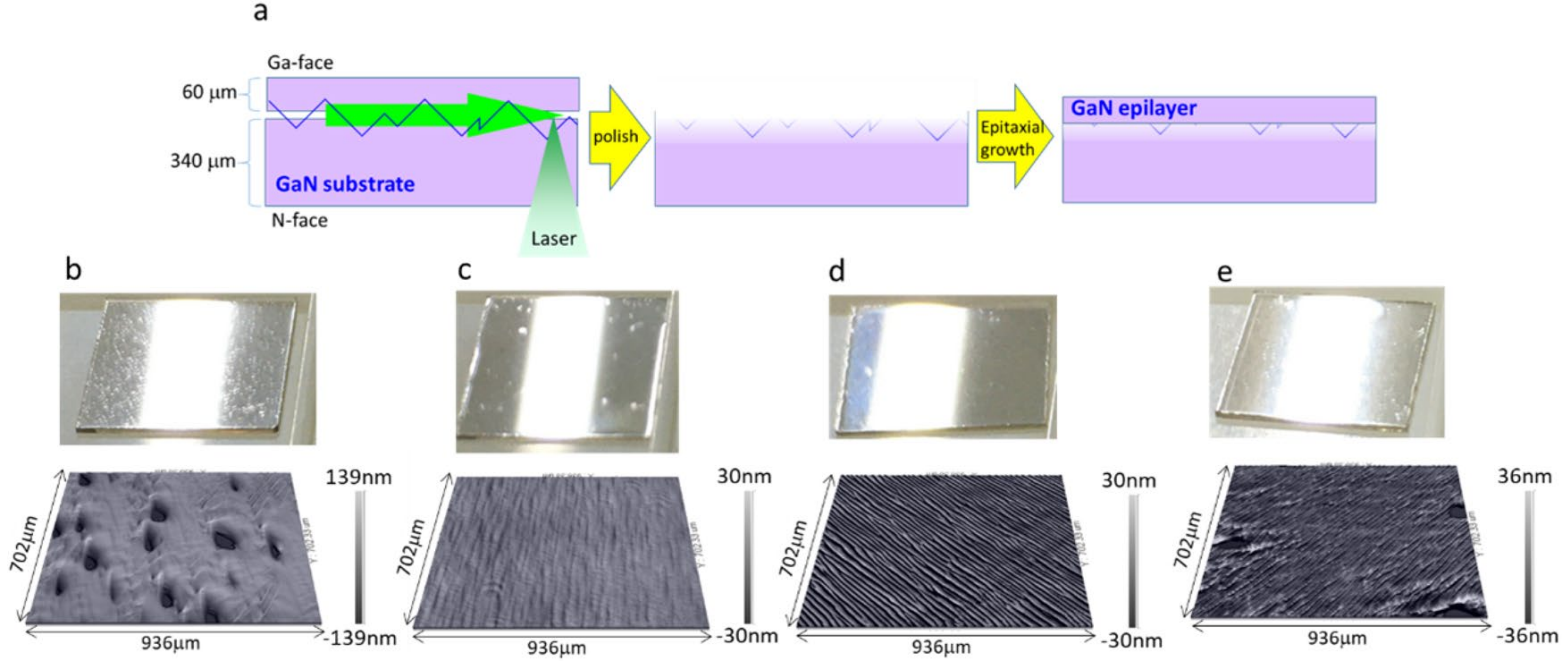
Photographs (upper) and white interference microscopy images (lower) of the samples after epitaxial growth. (**a**) Schematic image of experimental process. (**b**) Sample thinned by 18 µm. The arithmetic mean height of the area is 24 nm. (**c**) Sample thinned by of 47 µm. The arithmetic mean height of the area is 2.1 nm. (**d**) Sample thinned by 77 µm. The arithmetic mean height of the area is 7.2 nm. (**e**) Sample grown on commercially available epi-ready GaN substrate. The arithmetic mean height the of area is 8.0 nm.

**Figure 7:**
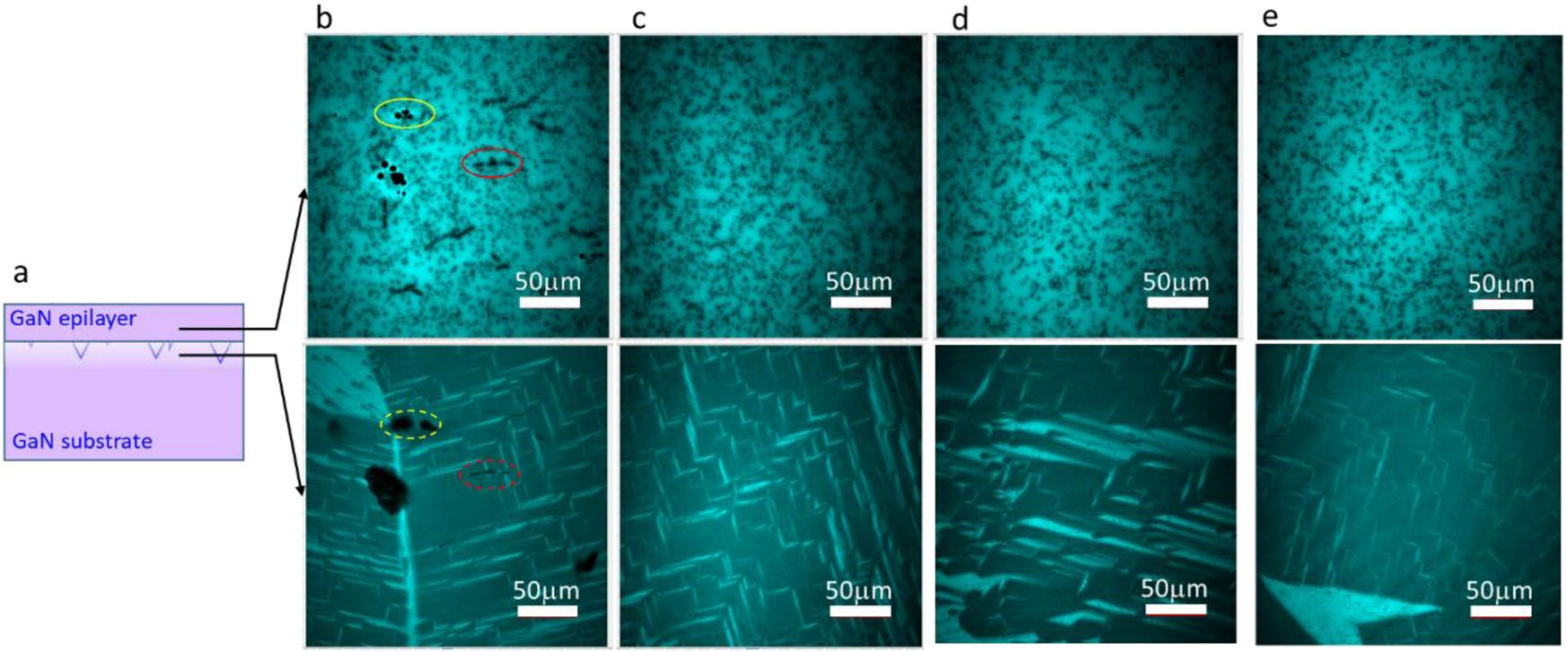
Multiphoton microscopy PL (365 nm) images of epitaxial layer (upper) and epitaxial layer/substrate interface (lower). (**a**) Cross-sectional schematic image showing the depth of observation. (**b**) Sample thinned by 18 µm. The red dashed circle indicates cracks, and the yellow dashed circle indicates holes on the substrate side immediately below the epitaxial layer/substrate interface. The generated dense dislocations (red circle) and voids (yellow circle) are observed in the epitaxial layer above the cracks and holes, respectively. The other dispersed black dots in the epitaxial layer are dislocations that originally existed in the substrate and propagated into the epitaxial layer. (**c**) Sample thinned by 47 µm. No dislocations are generated. (**d**) Sample thinned by 77 µm. No dislocations are generated. (**e**) Sample grown on commercially available epi-ready GaN substrate. No dislocations are generated.

To determine how the damage caused by laser slicing affects the crystal quality of the epitaxial layer grown on the sliced surface and the extent to which thickness should be decreased after laser slicing before epitaxial growth, we grew the epitaxial layer by metal organic vapor phase epitaxy (MOVPE) on the Ga surface of sliced samples whose thicknesses were decreased by various amounts by polishing them. The thickness of the epitaxial layer was 8 µm, assuming a typical vertical power device. In this study, we prepared samples with polishing amounts of 18 µm, 47 µm, and 77 µm and also a commercial epi-ready GaN substrate without slicing and polishing for comparison. In this experiment, we used 1 × 1 cm square samples. The results are shown in Fig. 6. As estimated above, when the amount of thickness decrease was less than 40 µm, the surface morphology of the epitaxial layer became rough. On the other hand, the roughness of the epitaxial layer surface was almost the same as that of the epitaxial layer grown on the commercially available epi-ready surface when the thickness decrease was more than 47 µm. With careful observation, they appear to have differences. However, the differences in the surfaces shown in Fig. 6c–e are within the range that appears as an in-plane distribution in the normal epitaxial growth of 2-in. wafers owing to its crystallinity distribution. Therefore, we do not think that the difference between the surface shown in Fig. 6c,d is due to laser damage or polishing. Figure 7 shows multiphoton microscopy PL images of the epitaxial layer/substrate interfaces of the samples. It is only on the substrate side of the sample thinned by 18 µm (Fig. 7b, bottom) that dark lines and dark areas are observed. These are probably because the crystalline parts of the damaged layer observed in Fig. 5 are mechanically and chemically weak, and cracks and holes were formed by the pressure and chemical agent of slurry applied for polishing. In the epitaxial layer, the epitaxial layer can be grown even on the cracks; however, clusters of dislocations are generated. On the holes, no epitaxial layer grows and voids remain. These are thought to cause the deterioration of the epitaxial surface morphology shown in Fig. 6.

## Conclusion

In this study, a sub-nanosecond 532 nm green laser equipped with LCOS-SLM was used slicing a GaN substrate. The laser slicing was carried out on the basis of a smart-cut-like principle, with almost no kerf loss, and the damage caused by laser irradiation had been shown to reach a depth of about 40 µm from the cutting surface. We also demonstrated that after removing the damaged layer of about 40 µm thickness, the laser-sliced surface is as suitable as a commercially available epi-ready GaN substrate for epitaxial growth.
